# Malignant glomus tumor of the duodenum Harboring novel FOXP1 and KDM5A mutations: a case report and literature review

**DOI:** 10.3389/fgene.2026.1761132

**Published:** 2026-05-26

**Authors:** Huiru Song, Yansong Li, Zeqi Liu, Shuai Yu, Yanan Wang, Si Liang

**Affiliations:** 1 Department of Pathology, Affiliated Hospital of Hebei University, Baoding, China; 2 Department of Pathology, The Second People’s Hospital of Hengshui, Hengshui, China; 3 School of Basic Medicine, HeBei University, Baoding, China; 4 Huisan Gene Technology (Shanghai) Co., Ltd, Shanghai, China; 5 Department of Anesthesiology, Affiliated Hospital of Hebei University, Baoding, China

**Keywords:** duodenum, FoxP1, KDM5A, malignant glomus tumor, next-generation sequencing, Notch signaling

## Abstract

**Background:**

Malignant glomus tumor (MGT) is an exceptionally rare mesenchymal neoplasm, accounting for less than 1% of all glomus tumors. Gastrointestinal involvement is unusual, and duodenal MGTs are exceedingly rare. Accurate preoperative diagnosis is difficult due to nonspecific clinical manifestations and overlapping histological features.

**Case Presentation:**

We report the case of a 47-year-old woman presenting with intermittent upper abdominal pain, aggravated after meals. Imaging revealed a heterogeneous enhancing lesion in the descending duodenum, and endoscopy showed a large ulcerative lesion. Initial biopsy suggested a glomus tumor. Whipple resection was performed, and histopathological examination demonstrated diffuse tumor growth with moderate nuclear atypia, atypical mitoses, and infiltrative extension into the mucosa and muscularis propria. Immunohistochemistry showed positivity for SMA, h-caldesmon, and synaptophysin, with negativity for CD117 and CgA. These findings, consistent with WHO 2019 diagnostic criteria, confirmed the diagnosis of duodenal MGT.

**Molecular Findings:**

Next-generation sequencing revealed two class III variants of uncertain significance: FOXP1 exon7 c.250C>T (p.P84S, VAF 22.27%) and KDM5A exon19 c.2801C>T (p.P934L, VAF 24.59%). Both mutations have not been previously reported in MGT. Biomarker analysis indicated low TMB (1.4 mutations/Mb) and microsatellite stability (MSS). The co-occurrence of FOXP1 and KDM5A mutations suggests potential involvement of NOTCH signaling dysregulation in MGT pathogenesis.

**Conclusion:**

This case represents a rare duodenal MGT confirmed by histopathology and immunohistochemistry, with novel FOXP1 and KDM5A mutations identified for the first time. These findings broaden the molecular spectrum of MGT and highlight the importance of integrating molecular profiling into the diagnosis and management of rare tumors, while surgical resection remains the cornerstone of therapy.

## 1 Introduction

Glomus tumors (GTs) are uncommon mesenchymal neoplasms that originate from the modified smooth muscle cells of the glomus body, typically located in the dermis of the extremities, particularly in the subungual regions of fingers and toes (Zhao et al., 2020; Mou et al., 2024). They account for less than 2% of all soft tissue tumors and are predominantly benign in nature. Visceral involvement of GTs is rare, with occasional cases reported in the stomach, respiratory tract, and other internal organs (Chen et al., 2020).

Malignant glomus tumors (MGTs) represent less than 1% of all GTs and are exceedingly rare (Alsahwan et al., 2021). Within the gastrointestinal tract, most reported cases arise in the stomach, while involvement of the duodenum is extremely uncommon (Deng et al., 2022). Owing to their rarity, MGTs of the intestine often present with nonspecific clinical manifestations and overlapping histopathological features, making accurate diagnosis particularly challenging. The current understanding of the clinicopathological spectrum, molecular alterations, and prognostic determinants of MGT remains limited, with most knowledge derived from sporadic case reports or small series (Bennett et al., 2015; Tan et al., 2015). Elucidating the molecular alterations underlying MGT is of paramount importance, as it may provide insights into tumorigenesis, reveal novel biomarkers for diagnosis or prognosis, and uncover potential targets for precision therapy. Given the limited efficacy of conventional chemotherapy and radiotherapy in MGT, the identification of recurrent or driver mutations could pave the way for personalized treatment approaches, especially in unresectable or metastatic settings.

Herein, we present a rare case of duodenal MGT with comprehensive clinicopathological evaluation. Importantly, next-generation sequencing (NGS) revealed two novel mutations in FOXP1 and KDM5A, both of which have not previously been reported in MGT. This finding not only enriches the molecular landscape of MGT but also raises new questions regarding its tumorigenesis and potential therapeutic targets. We also review the existing literature to contextualize the clinicopathological features and discuss the significance of these molecular findings.

## 2 Case presentation

A 47-year-old female patient presented with a 1-month history of intermittent upper abdominal pain, which was more pronounced after meals. She denied nausea, vomiting, fever, or other systemic symptoms. Initially, the patient self-medicated with omeprazole under the assumption of gastritis, but her symptoms persisted. She subsequently sought medical evaluation at our hospital.

**FIGURE 1:**
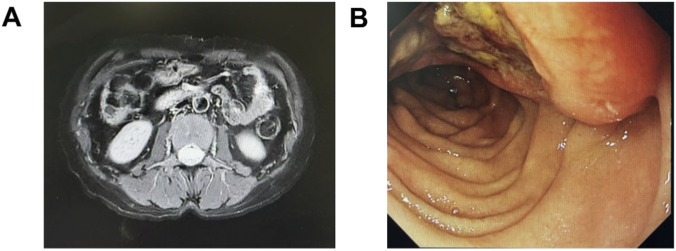
General condition of the patient. **(A)** Magnetic resonance imaging (MRI); **(B)** Endoscopic image of the descending part of the duodenum.

Magnetic resonance imaging (MRI) revealed an irregular lesion in the descending portion of the duodenum with heterogeneous moderate enhancement (Figure 1A). Endoscopic examination demonstrated a large ulcerative lesion occupying nearly half the circumference of the duodenal lumen, covered by necrotic exudates (Figure 1B). Multiple biopsies were obtained, and initial pathological evaluation suggested a glomus tumor. No lymphadenopathy or distant metastasis was detected on preoperative imaging.

## 3 Pathological examination

### 3.1 Biopsy Pathology

**FIGURE 2:**
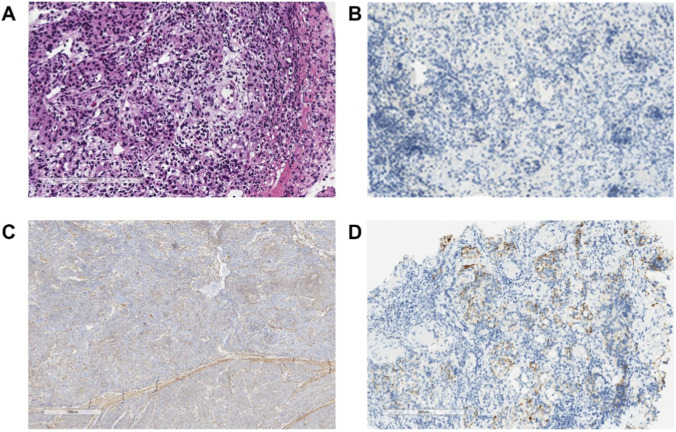
Biopsy Pathology images. **(A)** Microscopic photograph of biopsy tissue: Abnormal cell nests can be observed around the small blood vessels; **(B)** CK negative expression (CK^−^) in tumor sample; **(C)** Positive expression of SMA (SMA^+^) in tumor sample; **(D)** Positive expression of desmin (desmin^+^) in tumor sample.

Grossly, three small gray-white soft tissue fragments were obtained, measuring 0.05–0.2 cm in diameter. Microscopically, tumor cells were arranged in nests and trabeculae around proliferating small vessels. The cytoplasm appeared variably eosinophilic to clear, with centrally located round nuclei. Focal stromal inflammatory necrosis was observed (Figure 2A).

Immunohistochemistry (IHC) revealed: CK (−), CDX2 (−), CgA (−), Syn (+), Ki-67 index ∼20%, CK20 (−), ERG (vascular endothelium +), CK7 (−), CK8/18 (−), CD56 (−), EMA (−), desmin (+), calponin (−), S-100 (−), and SMA (+) (Figures 2B–D).

The morphological and immunophenotypic profile suggested a glomus tumor. However, due to limited biopsy material and atypical immunoreactivity, definitive assessment of malignancy could not be established. Further evaluation of the resected specimen was advised to confirm diagnosis and exclude malignant transformation.

### 3.2 Surgical Resection Specimen (Whipple procedure)

**FIGURE 3:**
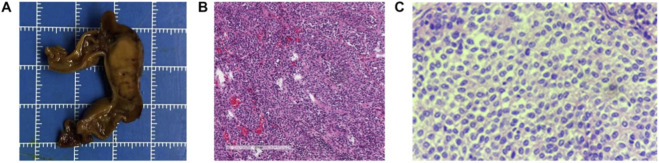
Biopsy Pathology images of Surgical Resection Specimen. **(A)** Gross examination: A mass is observed in the submucosa of the duodenum, with relatively well-defined borders. The cut surface appears grayish-white to light brown and is firm and tough in texture; **(B)** HE section: Diffuse sheets of tumor cells proliferate around capillaries. The tumor cells exhibit eosinophilic cytoplasm and hyperchromatic nuclei; **(C)** HE section: The tumor cells display clear cytoplasm, moderate nuclear atypia, prominent nucleoli, and mitotic figures are visible (400×).

Gross examination revealed a solid mass in the duodenum, measuring 3 × 3 × 2 cm, located approximately 1 cm from the pancreas. The cut surface appeared gray-white to tan, solid, and firm (Figure 3A).

Histologically, the tumor demonstrated diffuse sheets of cells encircling proliferating vasculature. The cytoplasm was variably clear to eosinophilic. Tumor nuclei showed moderate atypia, conspicuous nucleoli, and frequent mitotic figures, including atypical mitoses. Evidence of infiltrative growth through the duodenal mucosa and muscularis propria was noted (Figures 3B,C).

**FIGURE 4:**
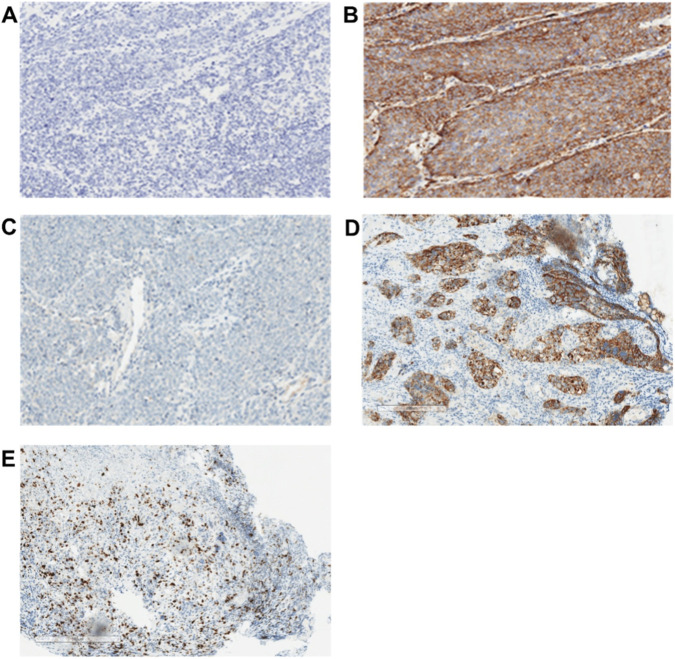
IHC images of Surgical Resection Specimen. **(A)** The tumor cells show negative expression for CgA (IHC, 200×); **(B)** The tumor cells show positive expression for H-caldesmon (IHC, 200×); **(C)** The tumor cells show negative expression for CD117 (IHC, 200×); **(D)** The tumor cells show positive expression for Syn (IHC, scale bar indicated at lower left); **(E)** The tumor cells exhibit a Ki-67 hotspot index of 20% (IHC, scale bar indicated at lower left).

**FIGURE 5:**
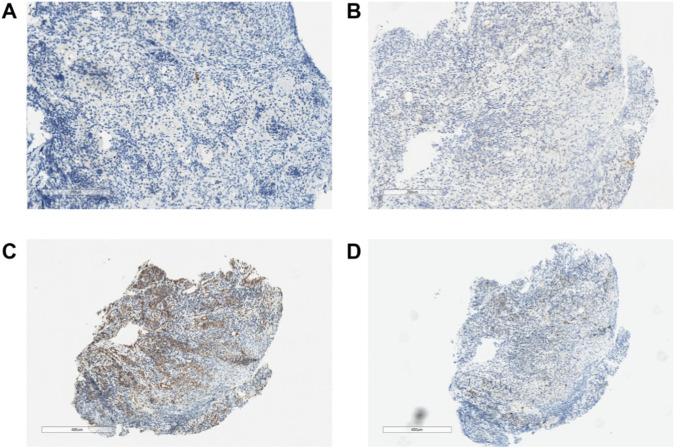
IHC images of Surgical Resection Specimen. **(A)** The tumor cells show negative expression for CgA; **(B)** The tumor cells show negative expression for EMA; **(C)** The tumor cells show positive expression for SMA; **(D)** The tumor cells show negative expression for S-100; IHC, scale bar indicated at lower left.

Immunohistochemistry showed: CgA (−), H-caldesmon (+), CD117 (−), Syn (+), Ki-67 labeling index ∼20%, CK (−), EMA (−), desmin (−), SMA (focally positive), CD34 (focal +), and S-100 (−) in hotspot areas (Figures 4, 5).

In line with the WHO 2019 classification of digestive system tumors, the diagnosis of malignant glomus tumor (MGT) was established, based on the combination of nuclear atypia, presence of atypical mitoses, and infiltrative growth pattern.

According to the WHO 2019 classification of tumors of the digestive system, the diagnosis of malignant glomus tumor requires a combination of features including deep location, tumor size >2 cm, moderate to marked nuclear atypia, presence of atypical mitotic figures, and infiltrative growth pattern (Nagtegaal et al., 2020). In the present case, the tumor measured 3 cm in maximum diameter, was located in the deep duodenal wall, exhibited moderate nuclear atypia with conspicuous nucleoli and atypical mitoses, and showed infiltrative extension into the duodenal mucosa and muscularis propria. These findings collectively satisfy the WHO 2019 diagnostic criteria for malignant glomus tumor, confirming the malignant nature of the neoplasm.

Final pathological diagnosis: Malignant glomus tumor of the duodenum, maximum diameter 3 cm, infiltrating the mucosa and muscularis propria, without definite vascular or perineural invasion, and no pancreatic involvement.

### 3.3 Molecular pathological findings

Next-generation sequencing (NGS) performed by SmartQuerier Gen Technology (Shanghai, China) revealed two class III variants of uncertain significance: FOXP1 exon7 c.250C>T (p.P84S, VAF 22.27%) and KDM5A exon19 c.2801C>T (p.P934L, VAF 24.59%). Both alterations have not previously been reported in malignant glomus tumors (MGT). Biomarker analysis showed a low tumor mutational burden (TMB, 1.4 mutations/Mb) and microsatellite stability (MSS), indicating limited sensitivity to immune checkpoint inhibitors.

The identification of FOXP1 and KDM5A mutations is of particular academic value. Both genes are closely associated with the regulation of the NOTCH signaling pathway, which plays an essential role in cell differentiation and tumorigenesis. FOXP1 has been reported to repress Jagged1 transcription and attenuate NOTCH activation, whereas KDM5A, a histone demethylase, can suppress NOTCH signaling and maintain neuroendocrine differentiation. The concurrent mutations in this case may suggest aberrant modulation of NOTCH signaling in MGT development. Although their precise biological role remains unclear, this is, to our knowledge, the first report of FOXP1 and KDM5A mutations in MGT, expanding the molecular spectrum of this rare tumor and highlighting the need for further functional investigations.

### 3.4 Postoperative and follow-up

Postoperative recovery was uneventful, and the patient has been followed for 12 months without evidence of recurrence or metastasis.

## 4 Discussion

Malignant glomus tumors (MGTs) are exceedingly rare neoplasms, accounting for less than 1% of all glomus tumors (Alsahwan et al., 2021). Involvement of the gastrointestinal tract is particularly uncommon, with most cases arising in the stomach, while duodenal MGTs have only rarely been reported. Because of their low incidence and nonspecific clinical manifestations, accurate preoperative diagnosis remains challenging. To contextualize our findings within the existing literature, we compared the clinicopathological characteristics of the present case with those of previously reported gastrointestinal malignant glomus tumors, as summarized in Table 1.

**TABLE 1:** Comparison of clinicopathological features Between the present case and previously reported gastrointestinal malignant glomus tumors.

Feature	Present case	Malignant gastric glomus tumor with heterochronous liver metastases (2025)	Malignant gastric glomus tumor with vascular invasion (2024)	Gastrointestinal glomus tumors: malignant ileal case from a 15-case series (2020)
Location	Duodenum	Stomach	Gastric antrum	Ileum
Age/Sex	47/F	36/F	49/M	50 (median)/(7M, 8F in cohort)
Tumor size	3 cm	2 cm	Not detailed (gastric mass found on CT)	1.5–3.0 cm (mean 2.3 cm, including benign)
Key findings(Mitosis/Ki-67/Invasion)	• Marked nuclear atypia • Atypical mitoses present • Ki-67 hotspot 20% • Infiltration into mucosa and muscularis propria	• Marked nuclear atypia • Mitotic count >5/5 mm^2^ • Ki-67 40% • Liver metastasis at 1 year post-surgery	• Moderate to severe nuclear atypia • Mitotic count >10/50 HPF • Vascular invasion present • No recurrence at 5 years post-surgery	• Marked cellular atypia • Mitotic count 5–6/HPF • Ki-67 approximately 70% • Liver metastasis at 15 months post-surgery
Immunohistochemistry(Positive/Negative)	Positive: SMA, h-caldesmon, Syn Negative: CD117, CgA, desmin, S-100	Positive: SMA, vimentin, Syn Negative: CD117, CD34, DOG-1, S-100	Positive: SMA, vimentin, Syn, h-caldesmon, Calponin Negative: (not detailed)	Positive: SMA, Collagen IV, caldesmon, calponin, Syn (weak+ in 12/15) Negative: (not detailed)
Molecular findings	FOXP1, KDM5A mutations (previously unreported in MGT)	NOTCH2 gene rearrangement BRAF V600E mutation (discussed as potential therapeutic target in malignant GT)	Not detailed	BRAF V600E mutation testing was negative in all cases
Treatment & Follow-up	Whipple procedure, no recurrence at 12 months	Local resection + RFA, alive with disease at 30 months	Distal gastrectomy, no recurrence at 5 years	Surgery, liver metastasis at 15 months
PMID	Present case	PMID: 40128164	PMID: 39121329	PMID: 31914530

The pathological diagnosis of MGT relies on the criteria proposed by the WHO 2019 classification of digestive system tumors, including deep location, tumor size >2 cm, moderate to marked nuclear atypia, atypical mitotic figures, and evidence of infiltrative growth (Nagtegaal et al., 2020). In the present case, the tumor measured 3 cm in maximum diameter, showed moderate nuclear atypia with conspicuous nucleoli, displayed atypical mitoses, and infiltrated the duodenal mucosa and muscularis propria, thereby meeting the diagnostic criteria for MGT.

Immunohistochemistry is essential for differential diagnosis. MGTs typically express smooth muscle markers such as SMA, h-caldesmon, and vimentin, while lacking expression of cytokeratin, desmin, S-100, and CD117 (Peng et al., 2024). This immunoprofile helps distinguish MGTs from other morphologically overlapping entities such as gastrointestinal stromal tumors (GISTs), which usually express CD117 and DOG-1; neuroendocrine tumors (NETs), which are positive for CgA, Syn, and CD56; and leiomyosarcomas, which exhibit strong SMA and desmin positivity along with more prominent necrosis and thick-walled vessels (Folpe et al., 2001).

A major novelty of this case lies in the molecular findings. NGS identified mutations in FOXP1 and KDM5A, both of which have not been previously reported in MGT. Their co-occurrence suggests a potential pathogenic role through aberrant NOTCH regulation in MGT. FOXP1 encodes a forkhead transcription factor that acts as a tumor suppressor in various malignancies by repressing Jagged1 and attenuating NOTCH signaling (Braccioli et al., 2017). KDM5A, a histone H3K4 demethylase, negatively regulates NOTCH target genes and maintains neuroendocrine differentiation in small cell lung cancer (Oser et al., 2019). Both mutations identified in our case are missense variants located in critical functional domains: FOXP1 p.P84S resides in the forkhead-associated domain, and KDM5A p.P934L lies within the JmjC demethylase domain. Computational prediction (SIFT, PolyPhen-2) suggests these alterations are likely deleterious, although functional validation is required. The convergence of these mutations on NOTCH signaling raises the possibility that dysregulation of this pathway contributes to MGT tumorigenesis, expanding the molecular landscape of this rare neoplasm. Although their biological significance remains to be validated, this molecular discovery expands the genomic landscape of MGT and highlights new avenues for mechanistic studies.

From a therapeutic perspective, complete surgical excision remains the mainstay of treatment, as chemotherapy and radiotherapy have shown limited efficacy in reported cases. The molecular profile of this patient indicated low TMB and MSS status, suggesting limited sensitivity to immune checkpoint inhibitors. Nonetheless, the identification of FOXP1 and KDM5A mutations raises the possibility of exploring targeted approaches and provides a rationale for incorporating molecular profiling into the management of rare tumors such as MGT.

## Data Availability

The original contributions presented in the study are included in the article/supplementary material, further inquiries can be directed to the corresponding authors.
